# Clinical impact of delaying initiation of adjuvant chemotherapy in patients with early triple negative breast cancer

**DOI:** 10.1007/s10549-023-07207-4

**Published:** 2024-01-19

**Authors:** Maria Eleni Hatzipanagiotou, Miriam Pigerl, Michael Gerken, Sophie Räpple, Verena Zeltner, Madeleine Hetterich, Peter Ugocsai, Elisabeth Christine Inwald, Monika Klinkhammer-Schalke, Olaf Ortmann, Stephan Seitz

**Affiliations:** 1 Department of Gynecology and Obstetrics, University Medical Centre Regensburg, Landshuterstraße 65, 93053 Regensburg, Germany; 2 Tumor Center Regensburg - Centre for Quality Management and Health Services Research, University of Regensburg, Regensburg, Germany; 3 Bavarian Cancer Registry, Regional Centre Regensburg, Bavarian Health and Food Safety Authority, Regensburg, Germany

**Keywords:** Triple negative breast cancer, Timing of adjuvant chemotherapy, Population-based cancer registry, Outcomes in TNBC, Routine practice data

## Abstract

**Purpose:**

The optimal time to initiation of adjuvant chemotherapy (TTAC) for triple negative breast cancer (TNBC) patients is unclear. This study evaluates the association between TTAC and survival in TNBC patients.

**Methods:**

We conducted a retrospective study using data from a cohort of TNBC patients diagnosed between January 1, 2010 to December 31, 2018, registered in the Tumor Centre Regensburg was conducted. Data included demographics, pathology, treatment, recurrence and survival. TTAC was defined as days from primary surgery to first dose of adjuvant chemotherapy. The Kaplan–Meier method was used to evaluate impact of TTAC on overall survival (OS) and 5-year OS.

**Results:**

A total of 245 TNBC patients treated with adjuvant chemotherapy and valid TTAC data were included. Median TTAC was 29 days. The group receiving systemic therapy within 22 to 28 days after surgery had the most favorable outcome, with median OS of 10.2 years. Groups receiving systemic therapy between 29–35 days, 36–42 days, and more than 6 weeks after surgery had significantly decreased median survival, with median OS of 8.3 years, 7.8 years, and 6.9 years, respectively. Patients receiving therapy between 22–28 days had significantly better survival compared to those receiving therapy between 29–35 days (p = 0.043), and patients receiving therapy after 22–28 days also demonstrated significantly better survival compared to those receiving therapy after more than 43 days (p = 0.033).

**Conclusion:**

Timing of adjuvant systemic therapy can influence OS in TNBC patients. Efforts should be made to avoid unnecessary delays in administering chemotherapy to ensure timely initiation of systemic therapy and optimize patient outcomes.

## Introduction

About 15% of breast cancers do not express estrogen (ER) or progesterone receptor (PgR) expression (≤ 1%) or show human epidermal growth factor receptor 2 (HER 2) overexpression or amplification [1, 2]. These triple negative breast cancers (TNBCs) usually have an aggressive tumor biology associated with a young age at diagnosis of less than 40 years [3]. Most TNBCs not only metastasize early in the course of the disease, but tend to develop prognostically unfavorable visceral and central nervous system metastases [4]. In comparison with other subtypes of breast cancer of the same stage, the survival rates of patients with TNBC are worse. The mortality rate of TNBC is 40% within the first 5 years after diagnosis [5]. Therapeutic options for patients with TNBC have been limited, but in the last years new therapeutic options are arising from the rapidly increasing knowledge on the pathogenesis and tumor biology [6–9]. Due to its specific molecular phenotype, TNBC is not sensitive to endocrine or molecular targeted therapy. Thus chemotherapy, in combination with immunotherapy depending on tumor stage, is currently the most important systemic therapy in early TNBC [9, 10]. Several randomized clinical trials have established that adjuvant and neoadjuvant administration of the same chemotherapy regimen yields in similar results in disease-free survival (DFS) and overall survival (OS) [11]. Modern treatment for TNBC consists in the application of neoadjuvant chemotherapy (NACT), since the neoadjuvant regimen provides an assessment of response to treatment and the opportunity to individualize therapeutical strategies [10, 12, 13]. The current standard of care for chemotherapy in the neoadjuvant as well as in the adjuvant setting in women with TNBC is an anthracycline and taxane based regimen [8, 14]. In recent years chemotherapy for TNBC is more likely to be given in the neoadjuvant setting than as an adjuvant but population-based data confirm that a relevant proportion of women with TNBC receive chemotherapy in the adjuvant setting, reflecting the clinical reality in the last years [1, 15, 16]. A recently published study [1] analyzed data to describe the current clinical practice regarding NACT in 94,638 patients with early breast cancer in Germany. They found that 31.8% of patients with TNBC received NACT, while 43% of the patients with TNBC received adjuvant chemotherapy. If neoadjuvant or adjuvant chemotherapy is administered, the duration should be 18–24 weeks [17]. Delays in time to surgery [18], time to adjuvant chemotherapy (TTAC) [18–23], time between neoadjuvant chemotherapy and surgery [24–26] as well as delays in starting NACT [27, 28] may impact patients outcome. The effect of TTAC in all breast cancer subtypes has been evaluated in several studies [18–23] with conflicting outcomes. Existing data suggest that a potential temporal impact is particularly important in TNBC, due to the aggressive tumor biology [20, 21]. We recently reported the impact of time to initiation of neoadjuvant chemotherapy (TTNC) on patient outcomes in a cohort of patients with TNBC [29]. The use of NACT in TNBC was increasing through the last years, but routine practice data shows that there is still a relevant proportion of patients with TNBC receiving adjuvant chemotherapy [1]. Therefore, we aim to clarify whether timing of adjuvant chemotherapy has an impact on survival in patients with early TNBC in a large population-based study using the Tumor Centre Regensburg registry database.

## Methods

### Study population and variables

**Fig. 1:**
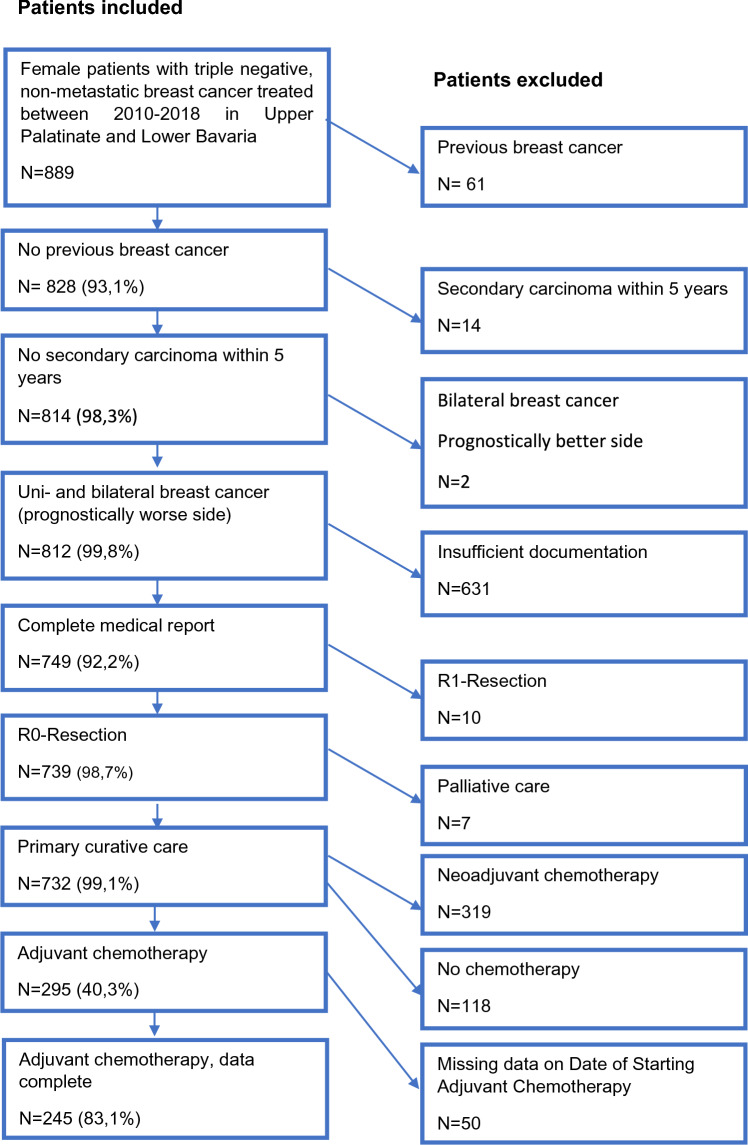
Depiction of the study collective

In this retrospective cohort study, clinical cancer registry data from the Tumor Centre Regensburg from patients with TNBC with a focus on diagnosis, therapy and recurrence were used for evaluation. A population of more than 2.2 million people including Upper Palatinate and Lower Bavaria is covered in this population-based regional cancer registry. Electronic sheets of documentation contain information about diagnosis, course of disease, therapies, and the complete follow-up of patients. These population‐based data originate from medical reports, pathology reports and follow‐up records. The Tumor Center Regensburg has been documenting tumor diseases in the Upper Palatinate and Lower Bavaria since 1991 and is integrated into the Institute for Quality Assurance and Health Services Research at the University of Regensburg. The population consisted of women living in Upper Palatinate and Lower Bavaria who have been diagnoses and treated with chemotherapy for TNBC and recorded by the Tumor Center Regensburg in the period from January 1, 2010 to December 31, 2018. Figure 1 describes inclusion and exclusion for the final study collective.

Patient information, including demographic characteristics and variables related to the cancer diagnosis and treatment, were abstracted from medical records by tumor registries as part of routine procedures. From the Tumor Center Regensburg we obtained the following patient characteristics: Data on tumor ER, PR, and Her2neu status, date of diagnosis of the primary tumor, age, last date of follow up, date of recurrence date, date of death, Charlson comorbidity index, site location, lymphatic vessel invasion, vein invasion, histopathological cancer stage, grading, Ki67, date of primary surgery and date of first application of chemotherapy.

### Statistical analysis

Patients who received adjuvant chemotherapy were categorized according to the time between primary surgery to the application of the first dose of adjuvant chemotherapy into six subgroups:  ≤ 14, 15–21, 22–28, 29–35, 36–42, ≥ 43 days. This interval was defined as TTAC. Descriptive statistics were used to evaluate the characteristics of the patient population according to TTAC subgroup. Follow-up was calculated using the reverse Kaplan–Meier method. Survival time was calculated in days from the date of breast cancer diagnosis to the date of last follow-up or the date of death. Patients who were alive at the study cutoff date of August 31, 2021 were censored on that date. The Tumor Center Regensburg regularly updates vital patient status information and active hospital follow-ups through linkages with state and national health offices and queries to the residents' registration offices. Recurrence-free survival was determined as the difference between the date of diagnosis of the primary tumor and the last living date, recurrence date, date of death or cut-off date. Univariable analyses of cumulative overall survival, cumulative recurrence rates, and recurrence-free survival were conducted using the Kaplan–Meier method, and the log-rank test was used for group comparisons. Mean survival time in years and a 5-year survival rate were analyzed, while median survival was not reached by the cut-off date. Data collection and statistical analysis were performed using IBM SPSS Statistic 25 (IBM Corporation, Armonk, NY), with p-values, and 95% confidence intervals (CI) calculated for each model. All tests were two-sided, and significance was set at p < 0.05.

## Results

We identified 15 011 patients with malignant neoplasms of the mammary gland that were coded by the „International Statistical Classification of Diseases and Related Health Problems” (ICD-10) Code C50 between January 1, 2010 to December 31, 2018. Among these we identified 889 female patients who were diagnosed with non-metastatic TNBC. Patients with previous breast cancer were excluded, which resulted in a population of 828 patients. Patients with a secondary carcinoma within the following 5 years and the prognostically more favorable side in bilaterally diseased patients were excluded. Incomplete or entirely missing documentation also led to exclusion. The final study cohort included 732 patients, of which 319 patients (43.6%) received NACT for TNBC, and 295 patients (40.3%) received adjuvant chemotherapy for TNBC. No chemotherapy was administered to 118 patients (16.1%), 63 of them refused any type of chemotherapy (8.6%) and in 49 patients (6.7%) the reason for renouncing chemotherapy was not documented, in 6 cases documentation on chemotherapy application was missing. 50 patients were excluded because of missing data on the exact date of starting adjuvant chemotherapy, so the TTAC could not be evaluated. Here, the results of the study subgroup of 245 patients with TNBC who were treated with adjuvant chemotherapy and had valid TTAC are reported. Out of the total patients, 172 women (70.2%) were postmenopausal and 73 (29.8%) were premenopausal at the time of TNBC diagnosis. The median TTAC was 29 days. The timing of adjuvant chemotherapy initiation after surgery was as follows: 34 patients (13.9%) started chemotherapy within 14 days after surgery, 47 patients (19.2%) between 15–21 days, 49 patients (20.0%) between 22–28 days, 55 patients (22.4%) between 29–35 days, 28 patients (11.4%) between 36–42 days, and 32 patients (13.1%) started chemotherapy 43 or more days after surgery. Patient, tumor, and treatment characteristics stratified by TTAC are shown in Table 1.

**Table 1:** Days from surgery to start of adjuvant systemic therapy (TTAC)

		Days from surgery to start of adjuvant systemic therapy (TTAC)													
		≤ 14		15–21		22–28		29–35		36–42		≥ 43		Total	
		N	(%)	N	(%)	N	(%)	N	(%)	N	(%)	N	(%)	N	(%)
Age at diagnosis	< 40	5	14,7	3	6,4	5	10,2	7	12,7	3	10,7	2	6,3	25	10,2
	< 50	4	11,8	13	27,7	9	18,4	10	18,2	4	14,3	2	6,3	42	17,1
	50–59	17	50,0	12	25,5	22	44,9	13	23,6	10	35,7	14	43,8	88	35,9
	60–69	5	14,7	9	19,1	7	14,3	11	20,0	6	21,4	7	21,9	45	18,4
	70 +	3	8,8	10	21,3	6	12,2	14	25,5	5	17,9	7	21,9	45	18,4
Menopausal Status	Premenopausal	11	32,4	17	36,2	17	34,7	17	30,9	7	25,0	4	12,5	73	29,8
	Postmenopausal	23	67,6	30	63,8	32	65,3	38	69,1	21	75,0	28	87,5	172	70,2
Co-morbidities	No	26	76,5	32	68,1	39	79,6	40	72,7	22	78,6	23	71,9	182	74,3
	Yes	5	14,7	10	21,3	7	14,3	11	20,0	5	17,9	9	28,1	47	19,2
	n.a	3	8,8	5	10,6	3	6,1	4	7,3	1	3,6	0	0,0	16	6,5
Stage	IA/B	17	50,0	18	38,3	21	42,9	21	38,2	15	53,6	10	31,3	102	41,6
	IIA	10	29,4	19	40,4	17	34,7	16	29,1	9	32,1	10	31,3	81	33,1
	IIB	6	17,6	5	10,6	5	10,2	9	16,4	1	3,6	6	18,8	32	13,1
	III	1	2,9	5	10,6	6	12,2	9	16,4	3	10,7	6	18,8	30	12,2
Tumor size	T1	19	55,9	23	48,9	29	59,2	26	47,3	19	67,9	12	37,5	128	52,2
	T2	14	41,2	20	42,6	17	34,7	24	43,6	7	25,0	17	53,1	99	40,4
	T3	1	2,9	4	8,5	3	6,1	4	7,3	2	7,1	2	6,3	16	6,5
	T4	0	0,0	0	0,0	0	0,0	1	1,8	0	0,0	1	3,1	2	0,8
Nodal Status	N0	27	79,4	35	74,5	33	67,3	36	65,5	21	75,0	18	56,3	170	69,4
	N1	6	17,6	8	17,0	10	20,4	13	23,6	6	21,4	9	28,1	52	21,2
	N2	1	2,9	2	4,3	5	10,2	3	5,5	1	3,6	4	12,5	16	6,5
	N3	0	0,0	2	4,3	1	2,0	3	5,5	0	0,0	1	3,1	7	2,9
Lymphatic vessel invasion	L0	14	41,2	26	55,3	26	53,1	41	74,5	23	82,1	24	75,0	154	62,9
	L1	12	35,3	18	38,3	21	42,9	13	23,6	5	17,9	8	25,0	77	31,4
	LX/n.a	8	23,5	3	6,4	2	4,1	1	1,8	0	0,0	0	0,0	14	5,7
Vein invasion	V0	25	73,5	41	87,2	38	77,6	49	89,1	28	100,0	32	100,0	213	86,9
	V1	0	0,0	4	8,5	7	14,3	5	9,1	0	0,0	0	0,0	16	6,5
	VX/n.a	9	26,5	2	4,3	4	8,2	1	1,8	0	0,0	0	0,0	16	6,5
Ki67	0–25	3	8,8	5	10,6	15	30,6	10	18,2	8	28,6	7	21,9	48	19,6
	> 25	28	82,4	41	87,2	34	69,4	43	78,2	19	67,9	24	75,0	189	77,1
	n.a	3	8,8	1	2,1	0	0,0	2	3,6	1	3,6	1	3,1	8	3,3
Type of surgery	BCT	31	91,2	40	85,1	43	87,8	44	80,0	21	75,0	28	87,5	207	84,5
	Mastectomy	3	8,8	7	14,9	6	12,2	11	20,0	7	25,0	4	12,5	38	15,5
	Total	34	100,0	47	100,0	49	100,0	55	100,0	28	100,0	32	100,0	245	100,0

*n.a.*  not available

At the time of analysis, 41 of 245 patients had died (16.7%). Overall, patients had an estimated mean OS of 9.3 years after surgery. The group that received systemic therapy within 22 to 28 days had the most favorable outcome, with a median OS of 10.2 years. The group that received systemic therapy within the first two weeks after surgery had a median OS of 9.9 years. However, for the groups that received systemic therapy between 29–35 days, 36–42 days, and more than 6 weeks after surgery, there was a significant decrease in median survival, with a median OS of 8.3 years, 7.8 years, and 6.9 years. An overview of mean survival time and 5-year survival can be seen in Table 2.

**Table 2:** Mean OS and 5-year survival rates according to TTAC

TTAC	n	Mean OS (years)	95% CI		
					5 year OS
≤ 14	34	9,881	8,991	10,771	87,6%
15–21	47	8,251	7,235	9,268	75,6%
22–28	49	10,207	9,446	10,969	90,8%
29–35	55	8,253	7,289	9,218	79,8%
36–42	28	7,762	7,053	8,471	85,7%
> 42	32	6,860	5,811	7,909	69,2%
Total	245	9,321	8,848	9,794	

**Fig. 2:**
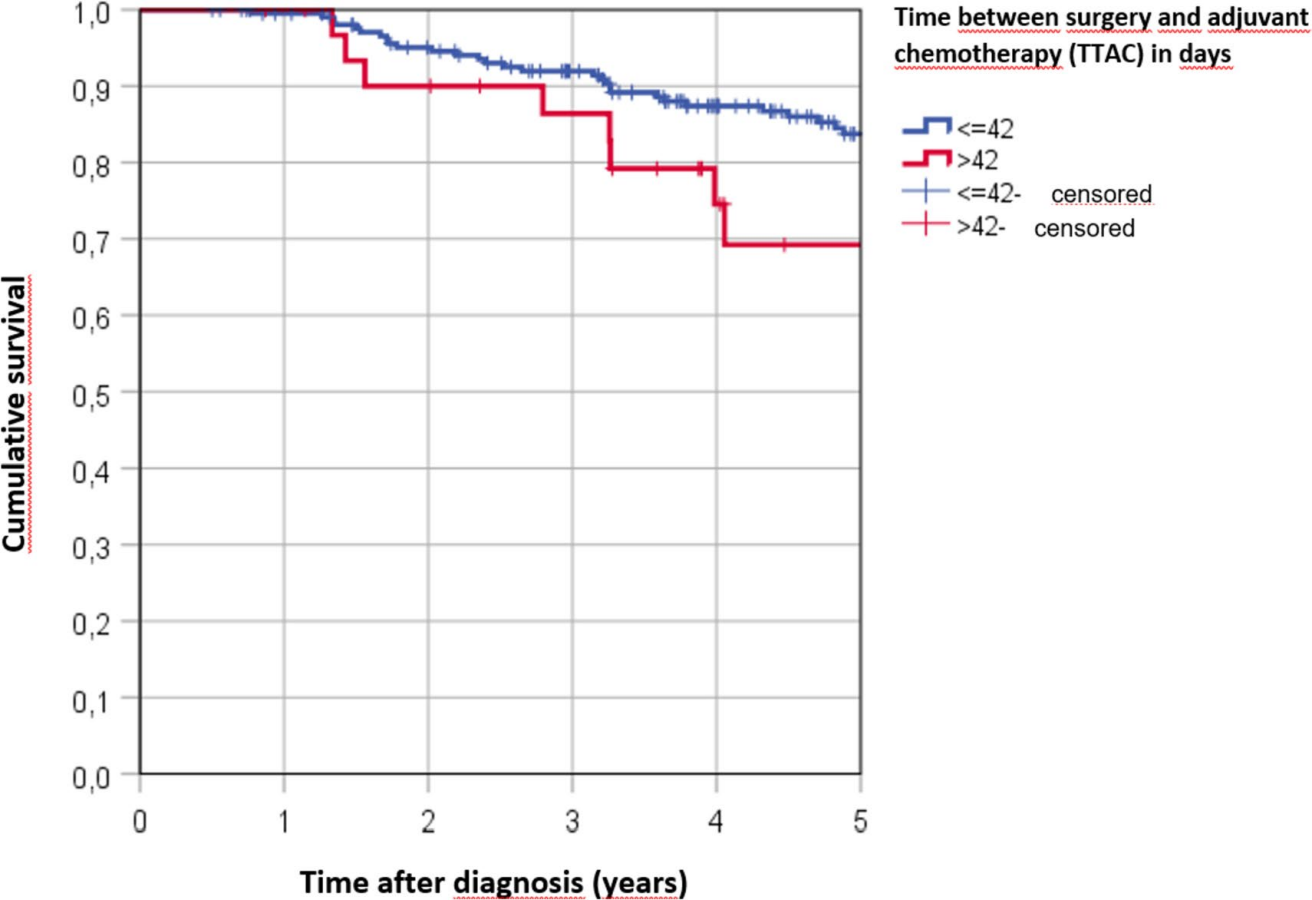
Kaplan–Meier 5 year survival rates according to TTAC

Most of the patients (n = 213, 86.9%) received adjuvant chemotherapy within 42 days after surgery, while only 32 patients (13.1%) started adjuvant chemotherapy after 42 days or more after surgery. Patients who started systemic therapy within 6 weeks after diagnosis were estimated to survive for 9.5 years, whereas patients who started adjuvant chemotherapy after 6 weeks survived only 6.9 years (see Table 2). Patients receiving therapy between 22–28 days had significantly better survival compared to those receiving therapy between 29–35 days (p = 0.043), and patients receiving therapy after 22–28 days also demonstrated significantly better survival compared to those receiving therapy after more than 43 days (p = 0.033). Figure 2 illustrates the Kaplan–Meier cumulative survival rates for TTAC ≤ 42 days versus TTAC > 42 days (p = 0,099).

## Discussion

This population-based study examined the impact of timing of adjuvant systemic therapy on OS in patients who underwent primary surgery for TNBC. TNBC is known for its aggressive nature, with early recurrence and limited treatment options beyond chemotherapy and recently immunotherapy [7, 8, 30, 31]. Therefore, timely initiation of adjuvant chemotherapy may be particularly crucial in this subtype of breast cancer. We observed that patients who received systemic therapy within 22–28 days after surgery had the most favorable outcome, with a median OS of 10.2 years, while patients who started adjuvant chemotherapy after 6 weeks had a lower median OS of 6.9 years. Patients who received therapy after 22–28 days demonstrated significantly better survival compared to those who received therapy after more than 43 days (p = 0.033).

The concept that commencing adjuvant chemotherapy early may have potential benefits is based on studies using animal models, which have shown increased levels of circulating growth factors and accelerated growth of metastases after the removal of the primary tumor [32] and on experiments using mice which demonstrated that administering a single dose of chemotherapy perioperatively or as an infusion within 3 days of surgery appears to be more effective compared to treatment given on day 7 after surgery [33]. Since the initial studies that demonstrated the potential benefits of early initiation of adjuvant chemotherapy in animal models, many subsequent studies have been conducted in breast cancer to investigate the optimal timing of adjuvant chemotherapy administration. Yu et al. [34] found that the effect of the influence of delayed initiation of adjuvant chemotherapy on breast cancer survival is subtype dependent. Patients with luminal-A tumors who received delayed chemotherapy had no increased risk of recurrence. In contrast, patients with luminal-B, triple-negative, or trastuzumab-untreated HER2-positive tumors showed decreased DFS because of delayed chemotherapy, highlighting the importance of timely initiation of chemotherapy in aggressive tumor subtypes. Biagi et al. conducted a systematic review and meta-analysis to assess the effect of delay in TTAC on survival in breast cancer patients. They involved two randomized trials and two cohort trails for OS, including data on 15.327 patients and their analysis demonstrated a 6% increase in the risk of death for each 4 week delay to initiation of adjuvant chemotherapy for breast cancer (HR, 1.06,95% CI 1.02 to 1.10 [19]. Different retrospective analysis confirmed that early initiation of adjuvant chemotherapy was associated with improved overall survival in patients with TNBC. However, the precise optimal time interval differs in the existing literature. A 4 weeks interval of starting adjuvant chemotherapy is supported by a retrospective analysis of Gagliato et al. [21]. They included 6,827 patients with breast cancer stages I to III who received adjuvant chemotherapy and categorized into three groups according to TTAC: ≤ 30, 31 to 60, and ≥ 61 days. Survival outcomes were estimated and compared according to TTAC and by BC subtype. Patients with TNBC tumors and those with HER2 –positive tumors treated with trastuzumab who started chemotherapy ≥ 61 days after surgery had worse survival (HR, 1.54,95% CI, 1.09 to 2.18 and HR, 3.09; 95% CI, 1.49 to 6.39, respectively compared with those who initiated treatment in the first 30 days after surgery.

In 2016 Chavez-MacGregor et al. conducted a retrospective population-based investigation to evaluate the timing of adjuvant chemotherapy initiation in patients with breast cancer. They observed that a 7-day delay in initiation of adjuvant chemotherapy increased the risk of death by 1% (HR 1.01; 95% CI 1.01–1.01). Their findings confirm the described subtype-dependent effect on OS of delaying adjuvant chemotherapy: Time to chemotherapy 91 or more days was associated with an increased risk in breast cancer death among patients with TNBC but had no significant effect among patients with hormone receptor– positive tumors. [20]. In 2018 Li et al. [22] retrospectively evaluated the effect of delayed adjuvant chemotherapy on relapse of TNBC in 331 patients. They found that delayed initiation of adjuvant chemotherapy beyond 60 days after surgery was associated with a significantly increased risk of relapse in TNBC patients [adjusted hazard ratio (HR of 2.39, 95% confidence interval (CI 1.13–5.07, P = 0.02]. A moderate delay (≤ 30 versus 31–60 days did compromise survival in lymph node positive patients. They conclude that initiation of adjuvant chemotherapy within 60 days would be the adequate time window for most TNBC cases, but an earlier initiation within 30 days might be more helpful for those TNBC patients with extremely high-risk factors.

However, few studies have reported conflicting results regarding the optimal timing of systemic therapy. For instance, Pomponio et. al in contrast published a retrospective analysis from a single-institution database in 2019 and found that TTAC was not significantly associated with DFS or OS in patient receiving chemotherapy for operable TNBC. Among the 724 patients included in their analysis, the median TTAC was 42 days. They observed that a TTAC > 56 days did not significantly impact DFS or OS (p = 0.27 and p = 0.21, respectively) compared to TTAC ≤ 31 days [23]. Other studies that did not find any association between TTAC and OS are relatively old and lack subtype analysis or differ in chemotherapy regimens from contemporary chemotherapy regimes. For instance, it's worth mentioning the nationwide clinical trial conducted by the Danish Breast Cancer Cooperative Group, which included 7501 breast cancer patients who received chemotherapy within 3 months of surgery between 1977 and 1999. The analyses were conducted in four groups of patients treated during weeks 1–3, 4, 5, and 6–13. The study found a similar prognosis for patients who started chemotherapy within 3 weeks after surgery compared to those who started chemotherapy up to 13 weeks after surgery [35]. However, it is not feasible to apply these results to the current era, as the prognosis of various breast cancer subtypes has significantly improved over time. Additionally, it is important to consider that the timing of initiation of highly effective and targeted therapies may also play a crucial role, given the increasing efficacy of these treatments. This notion is reinforced by more recent study findings as presented above. Taking our study and the mentioned studies into account, we suggest a 42 days interval after surgery for starting adjuvant chemotherapy in TNBC patients. A later initiation will impact survival outcomes. Known risk factors for delaying adjuvant systemic therapy are the presence of positive margins on pathology specimens and the need for re-surgery, age over 80, high comorbidity index, low socioeconomic status [36], breast reconstruction and nonprivate insurance [20]. Other possible risk factors may be wound healing after surgery, waiting for pathological result, postoperative case discussion, time for second opinion and the preparing of start of chemotherapy.

The strength of the present study lies in its utilization of a large population-based regional cancer registry with comprehensive and longitudinal data on diagnosis, therapy, and progression of TNBC in a population of over 2.2 million people. The study also employs rigorous statistical methods, including Kaplan Meier method, to examine the impact of delayed initiation of adjuvant chemotherapy on overall survival. However, there were several limitations in the study design that need to be acknowledged. For the analysis the time gap between the last surgery and the start of the adjuvant therapy was used. For patients who underwent more than one surgical procedure, the final surgery date before initiation of chemotherapy was used in this analysis. Time from biopsy to start of adjuvant chemotherapy is not available in our data. The specific question if the time interval between diagnosis and start of the adjuvant therapy has an impact on patient’s outcome, or if the time gap between last surgery and start of adjuvant therapy is the crucial point might be an interesting research question. The data obtained from the Tumor Centre Regensburg did not provide details on the type, dose, and duration of systemic therapy received by patients. Differences in the administered systemic therapy can have implications on OS and could potentially confound the results. Furthermore, the retrospective nature of registry-based studies and the use of statistical models to adjust for risk factors might not have accounted for all biases. It is challenging to conduct clinical trials to determine the optimal timing of adjuvant chemotherapy initiation due to ethical concerns, despite its significant clinical relevance. Therefore, population-based registries, as demonstrated in this study, provide a viable approach for answering this question.

The practical implications of the study findings are relevant for clinicians in making decisions about the optimal timing of systemic therapy after surgery. The results suggest that early initiation of systemic therapy within maximum 42 days after surgery is associated with better survival outcomes than delaying initiating of adjuvant chemotherapy 43 days or longer.

## Conclusion

In conclusion, our population-based study provides further evidence that the timing of adjuvant systemic therapy is a critical factor impacting OS in patients with TNBC who have undergone primary surgery. Our findings highlight the importance of timely initiation of adjuvant chemotherapy for optimal outcomes in TNBC patients. Specifically, starting systemic therapy within 42 days after surgery may be associated with better outcomes, while delaying therapy beyond 43 days may be associated with decreased survival. These results are consistent with the existing literature, which suggests that delaying adjuvant chemotherapy beyond 4–6 weeks after surgery may be associated with decreased OS, particularly in TNBC and other aggressive tumor subtypes. In daily clinical practice is crucial to prioritize timely initiation of adjuvant chemotherapy within the specified time frame, as it is feasible in most clinical scenarios. We should strive to avoid any unnecessary delays in administering chemotherapy to ensure optimal patient outcomes.

## Data Availability

The datasets generated during and analyzed during the current study are not publicly available due to maintenance and privacy of tumor registry data but are available from the corresponding author on reasonable request.
